# Assessment and Diagnosis of Mental Illness in EDs Among Individuals Without a Home: Findings from the National Hospital Ambulatory Care Survey

**DOI:** 10.5811/westjem.2021.7.51882

**Published:** 2021-11-05

**Authors:** Hijab Ahmed, Jeff A. Dennis

**Affiliations:** * Texas Tech University Health Sciences Center School of Medicine, Department of Emergency Medicine, Lubbock, Texas; † Texas Tech University Health Sciences Center, Department of Public Health, Lubbock, Texas

## Abstract

**Introduction:**

Homeless individuals lack resources for primary healthcare and as a result use the emergency department (ED) as a social safety net. Our primary objective in this study was to identify the differences between features of visits to United States (US) EDs made by patients without a home and patients who live in a private residence presenting with mental health symptoms or no mental health symptoms at triage.

**Methods:**

Data for this study come from the 2009–2017 National Health and Ambulatory Medical Care Survey, a nationally representative cross-sectional survey of ED visits in the US. We examined differences in waiting time, length of visit, and triage score among homeless patients, and privately housed and nursing home residents. We used logistic regression to determine the odds of receiving a mental health diagnosis. Residence, age, gender, race, urgency, and whether the person was seen in the ED in the previous 72 hours were controlled.

**Results:**

Homeless individuals made up less than 1% of all ED visits during this period. Of these visits, 47.2% resulted in a mental health diagnosis compared to those who live in a private residence. Adjusting for age, race, gender, triage score, and whether the person had been seen in the prior 72 hours, homeless individuals were still six times more likely to receive a mental health diagnosis despite reporting no mental health symptoms compared to individuals who lived in a private residence. Homeless individuals reporting mental health symptoms were two times more likely to receive a mental health diagnosis compared to privately housed and nursing home residents.

**Conclusions:**

Homeless individuals are more likely to receive a mental health diagnosis in the ED whether or not they present with mental health symptoms at triage. This study suggests that homelessness as a status impacts how these individuals receive care in the ED. Community coordination is needed to expand treatment options for individuals experiencing emergent mental health symptoms.

## INTRODUCTION

The United States Department of Housing and Urban Development estimates that 567,715 individuals experience homelessness on a single night.^1^ Homeless individuals may lack resources for primary healthcare and, as a result, use the emergency department (ED) as a de facto primary care physician as well as a social safety net.^2^ National survey data suggests homeless adults account for a disproportionate number of all ED visits relative to their population size.^3^

Previous research on homeless adults provides a demographic profile of homeless adults using the ED as well as characteristics of those visits. Homeless individuals who visit the ED are older, male, usually arrive via ambulance, and have a longer ED visit.^4^–^6^ Further, visits to the ED by older homeless adults are more often related to alcohol use and hospital admission whereas visits by younger homeless adults are related to psychiatric conditions and alcohol use.^2^ Homeless individuals who visited the ED for injuries were also more likely to be diagnosed with psychiatric or substance use disorders compared to non-homeless patients.^7^ A national dataset of aggregated ED visits from 2005–2015 revealed 28.4% of visits made by a homeless adult resulted in a psychiatric diagnosis.^3^ Further, homeless individuals with mental illness have higher frequency of 30-day readmissions compared to non-homeless individuals.^8^ No studies to date have compared homeless patients and privately housed residents on the presence or absence of mental health reasons for visit.

The intersection of homelessness and mental illness compounds the difficulties homeless individuals have in receiving healthcare. In this study we aimed to describe the prevalence of reported mental health symptoms and diagnosis by housing status in US ED visits. We asked two primary research questions: 1) Among those who report mental health symptoms at triage, do homeless individuals have different wait times, triage scores, or length of visit compared to the non-homeless; and 2) among individuals who report any or no mental health symptoms during ED triage, are homeless individuals more likely to receive a mental illness diagnosis upon discharge? We hypothesized that individuals without a home would be more likely to receive a psychiatric diagnosis irrespective of presenting reasons for visit.

## METHODS

### Study Design, Setting, and Study population

We used retrospective, cross-sectional data from the 2009–2017 National Hospital Ambulatory Medical Care Survey (NHAMCS), a nationally representative sample of ED visits in the United States collected annually by the National Center for Health and Statistics.^9^ The NHAMCS uses a three-stage probability sampling design where emergency service areas are sampled within hospitals and all emergency service areas of primary sampling stages. Primary sample stage consists of a sample of geographically defined areas. Randomly assigned EDs report their data for four weeks. This data is digitally recorded onto a patient record form by Census interviewers. The NHAMCS survey obtains data on patient and visit characteristics, clinician’s diagnosis (1–3 being the most relevant to the current ED visit and remaining diagnoses related to ongoing medical problems patient may have) health-related services, and treatments such as medications prescribed. The physician’s diagnosis was classified according to the International Classification of Disease, 9^th^ revision 9 (ICD-9) through 2015, and then shifted to ICD-10 in 2016.^10^,^11^ Patient and visit characteristics included age, gender, residence type, and race. Visit characteristics of interest included waiting time to see physicians and advanced practice providers (APP) in minutes; length of visit; and immediacy with which patient should be seen. We used data from 2009 forward because of a different NHAMCS coding scheme for triage score prior to 2009. Data used for the study were de-identified and publicly available and did not require review by the university’s institutional review board.

**Population Health Research Capsule**

What do we already know about this issue?

*Emergency department (ED) visits by homeless adults are disproportionate to the size of the homeless population with almost a quarter of visits related to mental health.*

What was the research question?

*What is the difference in ED visits between homeless adults and those who live in private residences?*

What was the major finding of the study?

*Homeless adults who do not present with mental health symptoms were six times more likely to receive a psychiatric diagnosis compared to privately housed residents and nursing home residents.*

How does this improve population health?

*This study suggests the homeless population may experience emergent mental health symptoms or that homelessness as a status impacts care received in the ED.*

### Independent Variables

Residence was coded into three categories: private residence, nursing home, and homeless. Homelessness was defined by NHAMCS as individuals who reported currently living in a homeless shelter or without a home. We excluded residences listed as “other,” “blank,” and “unknown.” Reason for visit was classified according to whether the patient reported any symptom related to mental health. Symptoms reported at triage were coded as mental health being a reason for visit for codes in the category “Symptoms Referable to Psychological and Mental Disorders” (codes 1100–1199), excluding code 1135 (disturbances of sleep) and “Mental Disorders” (codes 2300–2349).^12^ Further, we included intentional self-mutilation (5,818) and suicide attempt (5,820).^12^ Any mental health reason for visit included visits where at least one reported reason for visit was a mental health symptom. “Only mental health symptom” visits were defined as those visits where all reported reasons for visit were from one of the above codes. We included as control variables age, gender, race, and having used the ED in the prior 72 hours.

### Outcomes

Waiting time to see physicians and APPs in minutes, length of visit, and immediacy with which patient should be seen represent the first outcomes of interest. These variables were later used as controls in the examination of diagnosis at discharge. The dependent variable was diagnoses at ED discharge. These were further classified as either mental health diagnosis or non-mental health diagnosis. Mental health diagnosis was classified as any discharge diagnosis listed as ICD-9 codes 290–319 (excluding 310 – non-psychotic mental disorder due to brain damage), V62.84 (suicidal ideation), V71.09 (observation for suspected mental condition, or ICD-10 codes F01–F99.^10^,^11^ Within this group, substance use-related diagnoses were identified for comparison purposes, using codes 291, 292, and 303–305 in ICD-9 and codes F10–F19 in ICD-10.^10^,^11^

### Analysis

We performed statistical analysis using Stata 16.1 (StataCorp, LLC; College Station, TX) with population weighting to adjust for NHAMCS sampling design. Univariate analysis examines differences in triage score and wait time, and length of visit by place of residence using Wald tests to compare means. We used logistic regression to estimate odds of receiving a mental health diagnosis adjusting for residence, age, gender, race, urgency, and whether the person was seen in the ED in the previous 72 hours.

## RESULTS

The analysis sample for the nine-year NHAMCS study period included 183,085 adult ED visits. Of this sample, 12,384 (6.0%, weighted) cases included a person reporting a reason for visit that included mental health, and 18,365 (9.3%, weighted) were discharged with a mental health diagnosis. Individuals listed as “other” or “blank” for place of residence were dropped from analysis (N = 2,825; 1.5% weighted). We retained individuals for analysis with no listed reason for visit (“blank”) or no diagnosis in an attempt to best estimate the prevalence of mental health reasons for visit and mental health diagnosis in the sample of visits.

Homeless individuals represented slightly less than 1% of all ED visits during the time period but comprised a disproportionate number of visits for mental health reasons (Table 1). Over one-third of all homeless visits included some type of mental health reason for visit compared to about 5% of individuals who live in a private residence. Further, nearly half of all ED visits by homeless individuals resulted in a mental health diagnosis at discharge, compared to less than 10% of individuals who lived in a private residence. Homeless individuals who presented to the ED with only mental health symptoms received similar triage scores and had similar wait times compared to their counterparts who lived in a private residence (Table 2). However, the overall length of visit for homeless persons presenting with only mental health symptoms was nearly three hours longer (173 minutes) than for individuals who lived in a private residence. The average length of ED stay for these homeless individuals was over eight hours.

Table 3 shows the percentage of visits receiving a mental health diagnosis by type of residence and whether or not mental health symptoms were reported at admission. About three quarters of individuals living in a private residence who reported mental health symptoms at triage received a mental health diagnosis compared to about 7/8 of their homeless counterparts. Homeless individuals in this situation more often received a diagnosis related to substance use. Nearly 30% of homeless individuals who reported no mental health symptoms received a mental health diagnosis at discharge. Most of the mental health diagnoses among individuals reporting no mental health symptoms were related to substance use disorder.

Adjusting for age, race, gender, triage score, and whether the person had been seen in the prior 72 hours, homeless individuals were six times more likely to receive a mental health diagnosis despite reporting no mental health symptoms compared to individuals who live in a private residence (Table 4). Additionally, homeless individuals reporting any mental health symptoms at triage had two times higher odds of receiving a mental health diagnosis at discharge compared to those living in a private residence.

## DISCUSSION

The main purpose of this study was to determine how housing status is associated with triage score, wait time, and length of visit and whether individuals without a home were more likely to receive mental health diagnoses than individuals with a residence. We evaluated whether patients without a home were treated differently in terms of triage scores, wait times, and length of stay than those from private residences and nursing homes. Triage scores exhibited few differences by type of residence, for both mental health and non-mental health related symptoms. Patients without a home with any mental health symptoms waited longer to see a physician or APP compared to private residence patients. Given the similar triage scores, the long wait may be a result of the type of ED used, where individuals without a home visit public EDs with higher patient loads and longer wait time. However, NHAMCS stopped identifying the type of hospital in 2012 to preserve data security, so this explanation cannot be confirmed. Although NHAMCS does not provide insight into the reason for delay, the additional time where these patients are “boarded” in the ED may relate to limited options for inpatient beds, particularly among an uninsured or underinsured population.^14^ Future studies should explore how discharge disposition differs by place of residence among patients with mental health diagnosis. Although enacted after the data collection period, California Senate Bill 1152 mandates hospitals secure appropriate shelter or other resources for homeless individuals before discharge and, therefore, may further increase boarding time in the ED for this population.^15^

One of the main findings of this study was that individuals without a home who did not present with mental health reasons for visit were six times more likely to receive a mental health diagnosis than those living in a private residence. Our findings are in line with Lombardi et al who showed that individuals without a home were seven times more likely to receive a mental health diagnosis than non-homeless individuals comprising “other,” “private residence,” and “nursing home” residents.^3^ Furthermore, we showed that individuals without a home who present with mental health reasons for visit are still two times more likely to receive a mental health diagnosis than those living in a nursing home or private residence.

Whereas a high prevalence of mental health issues exists in the homeless population, stigma of homelessness may increase the likelihood of mental illness diagnosis, or physicians may be more hesitant to ascribe a diagnosis of mental illness in the ED for non-homeless patients as an avoidance of a stigmatizing label.^13^ Also, there is no variable accounting for past medical history; so it is possible that individuals without a mental health reason for visit have mental illness that is documented in the clinician’s diagnoses. Care in the ED is designed for stabilization of acute health episodes and is far from the ideal location for treatment of chronic mental illness in the population. Lack of community resources for acute mental health care, as well as long-term management of mental illness, may contribute to overuse of the ED for mental health reasons.^16^ Community programs that create more comprehensive services, including continuity of care and non-ED crisis services, and law enforcement collaborations that reduce prevalence of persons with mental illness in both the ED and jail may alleviate the problem; however, such programs require meaningful collaboration across agencies.^16^

## LIMITATIONS

The NHAMCS is a cross-sectional dataset of ED visits and does not identify patients across multiple encounters. Therefore, the exact prevalence of homelessness or mental health symptoms in this community is unknown. Homeless individuals who are chronic consumers of ED services may be treated differently because they are known to most staff. Although we cannot adjust fully for this possibility, NHAMCS provides some context with the variable for “visit made within the past 72 hours.” In addition, there is no separate variable addressing past psychiatric history for each visit; therefore, it is possible that visits made by individuals without a home who do not present with a mental health reason for visit may receive a mental health diagnosis based on prior history. This was addressed by comparing individuals without a home who do present with mental health symptoms to individuals residing in a private residence or nursing home.

From a clinical perspective, the NHAMCS data do not provide the context or nuanced information that emergency clinicians use daily in the management of patients with mental health symptoms. As a population-based study, the aggregation of individuals by mental health symptoms and diagnosis is intended only for broad characterization of how this population is managed on a national level and may not match up with the individual experience of some emergency physicians or APPs.

## CONCLUSION

This study revealed that homeless individuals in the ED were more likely to receive a mental health diagnosis whether they reported mental health symptoms at triage or not. Further, homeless patients presenting with mental health symptoms experienced longer stays and wait times than patients living in a private residence who presented with mental health symptoms. The prevalence of mental health symptoms and diagnoses among homeless individuals in the ED demonstrates the need for ongoing efforts to improve healthcare access and continuity of care in this population.

## Figures and Tables

**Table 1:** Sample characteristics by residence type.

	Private residence N (weighted %)	Nursing home N (weighted %)	Homeless N (weighted %)	P value
Overall	167,669 (96.76)	4,215 (2.38)	2,198 (0.86)	
Female	96,475 (58.06)	2,571 (60.80)	581 (27.37)	<0.001
Male	71,194 (41.94)	1,644 (39.20)	1,617 (72.63)	
Race/ethnicity				
NH White	102,775 (61.84)	3,252 (77.78)	1,145 (56.08)	<0.001
NH Black	36,820 (22.53)	580 (13.47)	630 (24.40)	
Hispanic	22,002 (12.76)	274 (6.64)	328 (14.67)	
Other	6,072 (2.87)	109 (2.11)	95 (4.85)	
Seen in ED past 72 hours				
No	141,199 (95.56)	3,552 (95.93)	1,764 (89.97)	<0.001
Yes	7,039 (4.44)	145 (4.07)	238 (10.03)	
Mental health symptoms at triage				
Any	9,811 (5.35)	592 (13.61)	817 (34.40)	<0.001
None	157,858 (94.65)	3,623 (86.39)	1,381 (65.60)	
Only mental health symptoms at triage	3,811 (1.92)	224 (5.45)	346 (12.78)	<0.001
Any MH diagnosis at discharge	15,308 (8.71)	498 (11.37)	1,111 (47.02)	<0.001

*NH*, non-Hispanic, *ED*, emergency department; *MH*, mental health.

**Table 2:** Mean differences in age and emergency department characteristics by housing status.

	Private residence Mean (SD)	Homeless Mean (SD)	P value	Nursing home Mean (SD)	Homeless Mean (SD)	P value
Age	45.3 (19.2)	43.8 (16.2)	<0.001	76.1 (16.0)	43.8 (16.2)	<0.001
Any mental health symptoms						
Triage score	3.0 (0.9)	3.0 (1.1)	0.089	2.8 (0.8)	3.0 (1.1)	0.097
Wait time (mins)	35.3 (70.9)	52.7 (151.6)	0.013	35.5 (97.8)	52.7 (151.6)	0.042
Length of visit (mins)	315.0 (447.7)	492.8 (749.5)	<0.001	297.9 (329.0)	492.8 (749.5)	<0.001
Only mental health symptoms						
Triage score	2.8 (0.9)	2.8 (1.1)	0.820	2.8 (0.8)	2.8 (1.1)	0.833
Wait time	36.1 (77.2)	63.2 (211.8)	0.087	24.7 (57.4)	63.2 (211.8)	0.018
Length of visit	334.4 (453.5)	507.6 (614.5)	<0.001	327.6 (306.3)	507.6 (614.5)	<0.001
No mental health symptoms						
Triage score	3.3 (0.8)	3.4 (1.0)	0.606	3.0 (0.8)	3.4 (1.0)	<0.001
Wait time	38.1 (70.0)	48.8 (106.3)	0.002	29.9 (61.9)	48.8 (106.3)	<0.001
Length of visit	204.2 (244.5)	276.0 (403.2)	<0.001	256.2 (249.9)	276.0 (403.2)	0.238

*ED*, emergency department; *SD*, standard deviation; *mins*, minutes.

**Table 3:** Percent receiving a mental health diagnosis at discharge by residence and reason for visit.

	Private residence N (weighted %)	Homeless N (weighted %)	P value	Nursing home N (weighted %)	Homeless N (weighted %)	P value
Presenting with only MH symptoms						
Received any MH diagnosis	3,018 (76.1)	302 (87.7)	<0.001	104 (38.4)	302 (87.7)	<0.001
Received any SUD diagnosis	957 (25.1)	151 (43.8)	<0.001	8 (5.3)	151 (43.8)	<0.001
Received non-SUD MH diagnosis	2,061 (50.9)	151 (44.0)	0.104	96 (33.1)	151 (44.0)	0.114
Presenting with no MH symptoms						
Received any MH diagnosis	8,697 (5.7)	432 (29.2)	<0.001	271 (8.0)	432 (29.2)	<0.001
Received any SUD diagnosis	5,018 (3.4)	336 (24.2)	<0.001	18 (0.4)	336 (24.2)	<0.001
Received non-SUD MH diagnosis	3,679 (2.2)	96 (5.1)	0.009	253 (7.6)	96 (5.1)	0.058
Presenting with only MH symptoms						
Received any MH diagnosis	3,018 (76.1)	302 (87.7)	<0.001	104 (38.4)	302 (87.7)	<0.001
Received any SUD diagnosis	957 (25.1)	151 (43.8)	<0.001	8 (5.3)	151 (43.8)	<0.001
Received non-SUD MH diagnosis	2,061 (50.9)	151 (44.0)	0.104	96 (33.1)	151 (44.0)	0.114
Presenting with no MH symptoms						
Received any MH diagnosis	8,697 (5.7)	432 (29.2)	<0.001	271 (8.0)	432 (29.2)	<0.001
Received any SUD diagnosis	5,018 (3.4)	336 (24.2)	<0.001	18 (0.4)	336 (24.2)	<0.001
Received non-SUD MH diagnosis	3,679 (2.2)	96 (5.1)	0.009	253 (7.6)	96 (5.1)	0.058

*MH*, mental health; *SUD*, substance use disorder.

**Table 4:** Logistic regression of receiving a mental health diagnosis at discharge based on presence or absence of mental health symptoms at triage (Source: NHAMCS 2009–2017).

	No MH symptoms at triage OR (95% CI)	MH symptoms at triage OR (95% CI)
Residence type		
Private	Reference	
Nursing home	**1.7 (1.3,2.3)**	**0.4 (0.2,0.6)**
Homeless	**6.7 (5.3,8.5)**	**2.2 (1.2,4.0)**
Age	**1.0 (1.0,1.0)**	**1.0 (1.0,1.0)**
Male	**1.3 (1.2,1.4)**	0.9 (0.7,1.2)
Race/ethnicity		
NH White	(Reference)	
NH Black	**0.8 (0.7,0.9)**	0.9 (0.6,1.3)
Hispanic	**0.8 (0.6,0.9)**	0.8 (0.5,1.3)
Other	1.0 (0.8,1.3)	0.5 (0.3,1.0)
Triage score	**0.8 (0.7,0.8)**	**1.2 (1.0,1.4)**
Seen in past 72 hours	1.2 (1.0,1.4)	0.6 (0.4,1.0)

Bolded values indicate P<0.05.

*MH*, mental health; *OR*, odds ratio; *CI*, confidence interval; *ref*, reference; *NH*, non-Hispanic: *NHAMCS*, National Hospital Ambulatory Medical Care Survey.
